# 3D Printed Silicones with Shape Memory

**DOI:** 10.1038/s41598-017-04663-z

**Published:** 2017-07-05

**Authors:** Amanda S. Wu, Ward Small IV, Taylor M. Bryson, Emily Cheng, Thomas R. Metz, Stephanie E. Schulze, Eric B. Duoss, Thomas S. Wilson

**Affiliations:** 1 Lawrence Livermore National Laboratory, Materials Engineering Division, Livermore, CA 94550 USA; 2 Lawrence Livermore National Laboratory, Materials Science Division, Livermore, CA 94550 USA; 3 Department of Energy’s National Security Campus, managed by Honeywell, Materials Engineering, Kansas City, MO 64147 USA

## Abstract

Direct ink writing enables the layer-by-layer manufacture of ordered, porous structures whose mechanical behavior is driven by architecture and material properties. Here, we incorporate two different gas filled microsphere pore formers to evaluate the effect of shell stiffness and T_g_ on compressive behavior and compression set in siloxane matrix printed structures. The lower T_g_ microsphere structures exhibit substantial compression set when heated near and above T_g_, with full structural recovery upon reheating without constraint. By contrast, the higher T_g_ microsphere structures exhibit reduced compression set with no recovery upon reheating. Aside from their role in tuning the mechanical behavior of direct ink write structures, polymer microspheres are good candidates for shape memory elastomers requiring structural complexity, with potential applications toward tandem shape memory polymers.

## Introduction

The 3D printing process employed in this work, also known as direct ink writing (DIW), enables the layer-by-layer manufacture of ordered, porous structures whose mechanical behavior is driven by architecture and material properties. We pursue hierarchical porosity as a means of lightweighting, tailoring mechanical response and introducing functionality into 3D printed silicones. Hierarchical porosity is achieved by a combining printed structural porosity with intrastrand porosity, obtained by adding hollow, gas-filled microspheres to the ink. Aside from their role in tuning the mechanical behavior of 3D printed architectures, polymer microspheres are good candidates for shape memory applications requiring structural complexity with the ability to achieve both open or closed cell porosity. Here, for the first time, we demonstrate that shape memory can be achieved in 3D printed porous elastomers simply by the addition of polymer microspheres with controlled shell glass transition temperatures.

Process development and fabrication of stochastic elastomeric foams is driven by diverse applications requiring advanced structural performance facilitated by both closed cells (e.g., shock absorption, acoustic damping and thermal insulation) and open cells (e.g., biocompatible membranes, tissue engineering scaffolds, semipermeable membranes for materials separation and food processing)^1–6^. This application space has benefitted from structural control, enabled by a family of emerging technologies, broadly known as 3D printing. Recently, 3D printing of silicones has been used to create mechanical energy absorbing materials with negative stiffness^7^, vascularized tissue constructs^8^, stretchable sensors^9^, soft robotics^10^, and shape morphing materials^11^. These advances are made possible by the flexible and stretchable nature of silicone elastomers, combined with the unique structural and compositional control enabled via 3D printing.

Applications benefitting from structurally engineered porosity accommodated by 3D printing include engineered tissue scaffolds^12^, photolithographic patterned nanowire growth for tailored electronics^13, 14^, and nanolithography metamaterials with a negative refractive index for cloaking and superlensing applications^15^, engineered with unit cells smaller than the wavelength of light^16^. In addition to their predictability, repeatability and potential for architectural complexity, ordered porous structures are desirable over stochastic foams from a long-term mechanical performance standpoint, due to their minimization of local stress concentrations which can result in localized material failure^17^.

Further spatial and temporal control can be achieved by 3D printing with shape memory polymers^18^. Since their development, in the 1960’s^19^, polymers with shape memory behavior^20^ have found applications in self-repairing components^21^, high performance textiles^22^, and surgical medicine^23^. More recent advancements in this field include shape memory polymers with elastomeric behavior at elevated temperatures^24^ and very large strain and energy storage capacities have been reported^25–27^.

In the field of net-shape processing, shape memory behavior can provide enhanced tunability and functionality to 3D printed objects, enabling controlled structural deformation to occur post-processing. Imbuing 3D printed objects with the ability to change their configuration in response to external stimuli is colloquially known as “4D printing”, where the fourth dimension is time^28^.

Our 3D printing approach enables property-specific tailoring, resulting in mechanical metamaterials that are tuned with constituent material behavior, porosity and structure^7^. Examples of 3D printed metamaterials can be found in ceramic^29–31^ and metallic^32, 33^ hierarchical lattice structures^34^ with mechanical behavior outside that of conventionally processed materials achieved through tuning levels of hierarchy, porosity and material constituents.

## Results

### 3D printed materials

**Figure 1:**
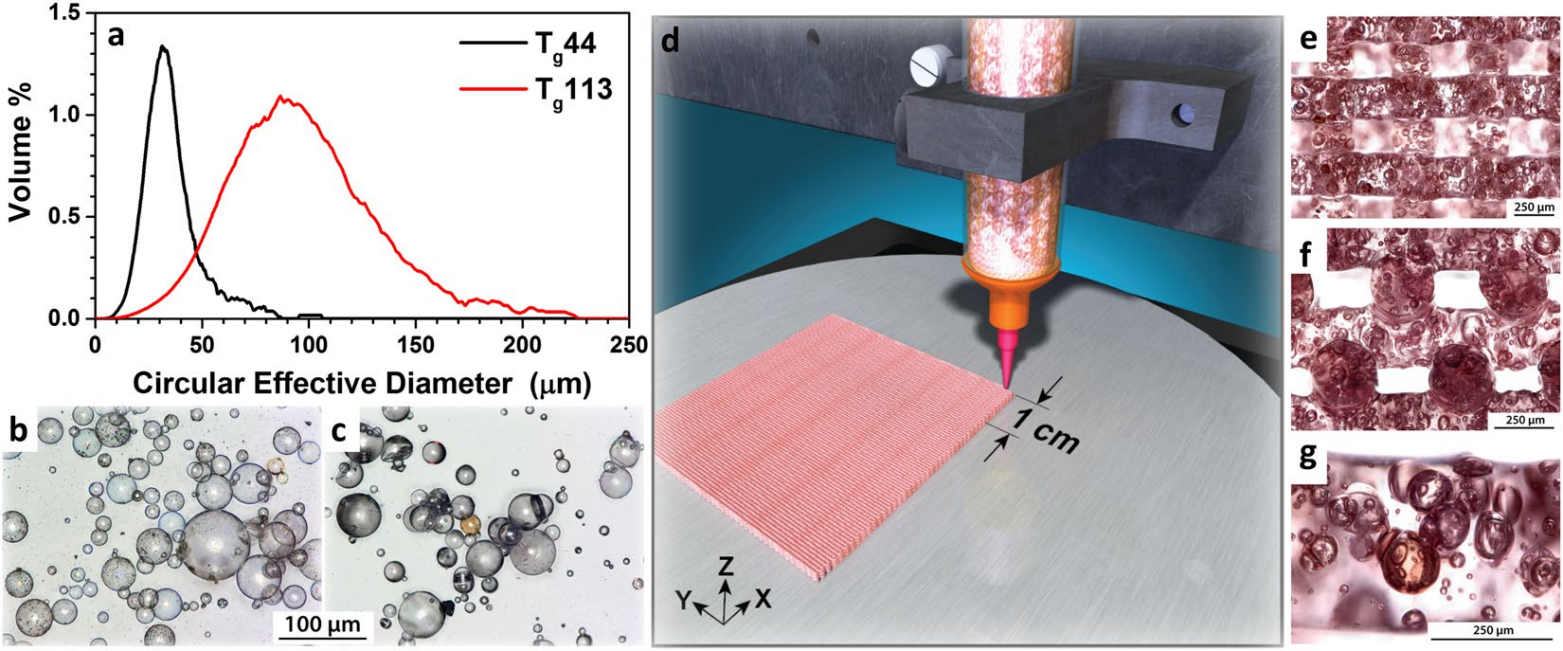
(**a**) Microballoon diameter size distribution, optical microscopy images of (**b**) T_g_44 and (**c**) T_g_113 microballoons, (**d**) schematic illustration of our 3D printing process, optical microscopy images of printed silicones with microballoons showing (**e**) x-y view, (**f**) x-z view and (**g**) high magnification image of (**f**) showing 25 vol% microballoons in a printed filament.

In the 3D printing process, viscoelastic inks with highly controlled rheological behavior are extruded through a microscale nozzle or die, resulting in the layer-by-layer building of programmable architectures whose complexity is controlled by strand size and spanning distance over gaps in the underlying layers^35^. The former is influenced by the applied pressure, die geometry and rheological response of the resin, while the latter is a function of gel strength, deposition speed, shear rate and resin density^36^. Here, we pursue intrastrand porosity using a silicone based ink comprised of polymeric shell, gas filled microspheres or microballoons to further enhance the compressibility of porous elastomeric structures. Figure 1a–c illustrates the two different gas filled microballoon pore former particle size distributions used to evaluate the effect of shell stiffness and glass transition temperature, T_g_ (44° and 113°, see Figure S1), on compressive behavior and shape memory in our printed structures (Fig. 1d–g).

### Rheological behavior

**Figure 2:**
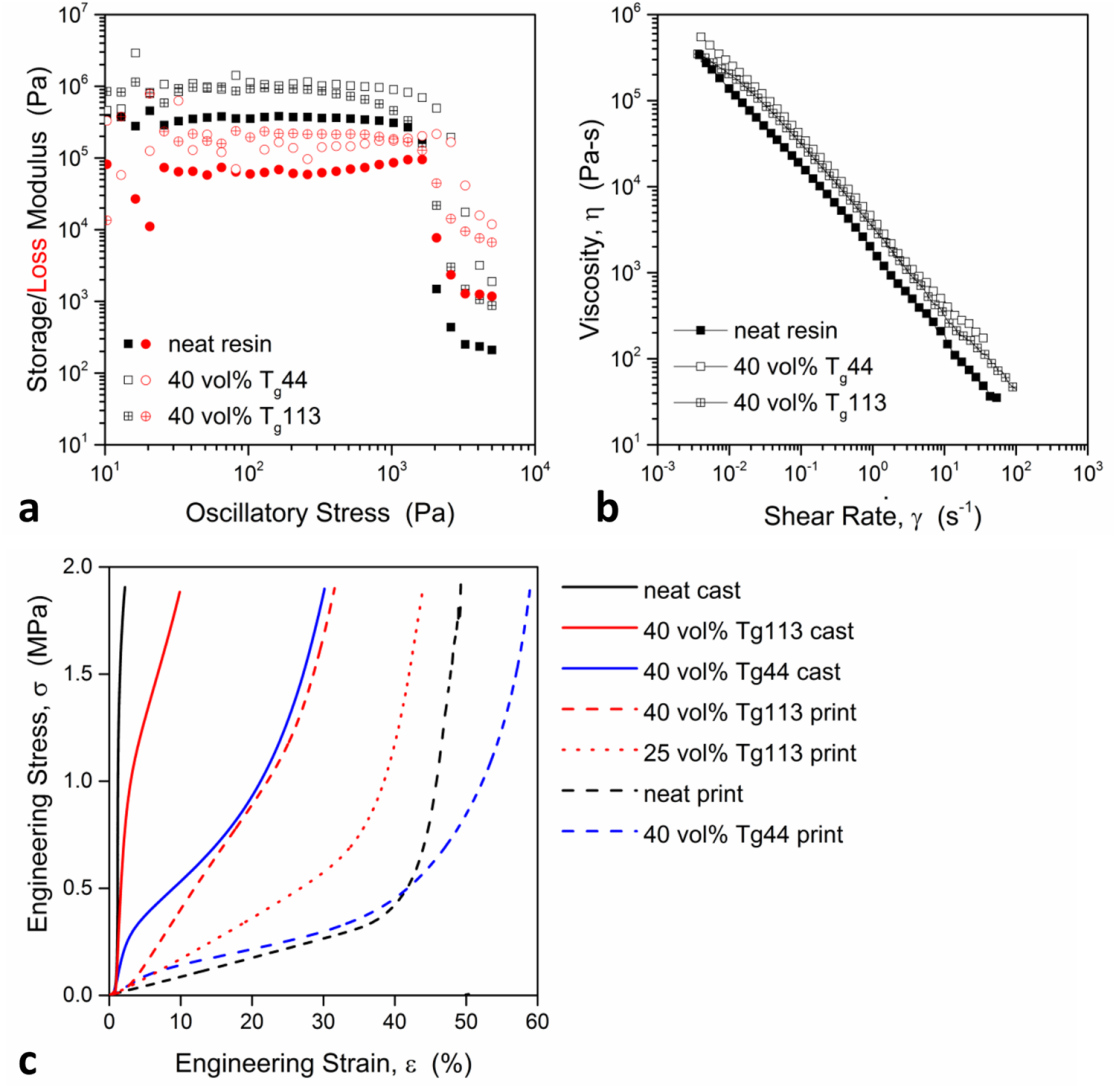
Effect of microballoons on (**a**) rotational oscillatory response, (**b**) continuous flow behavior of the ink, and (**c**) response to compressive loading of the cast and printed structures.

To achieve optimal elastomeric flow behavior for our composite inks, we performed stress-controlled rotational rheology experiments using a variety of conditions with a typical microballoon loading of 40 vol%. Under oscillatory flow at a frequency of 10 Hz (Fig. 2a), the effect of microballoon addition manifests as an increase in storage and loss moduli, accompanied by a slight increase in yield stress in the case of the T_g_44 resin, while maintaining printability. No permanent die swell was observed through either measurement of printed strands or *in situ* measurement of flow near the die exit. This is attributable to power law behavior due to wall slip/plug flow (Fig. 2b), indicating that no configurational entropy is recovered upon nozzle exit.

Subtle influences of the pore former size distribution and volume loading on rheological behavior are observed; however, printability and structural repeatability is minimally affected. The DIW process, illustrated in Fig. 1d, highlights the potential of gas filled microballoons to substantially reduce the printed strand density, which can lead to enhanced strand spanning capability^37^.

### Mechanical response

Gas filled microballoons provide a means of tuning the mechanical performance of 3D printed elastomers, beyond architecture; the lattice is limited by nozzle diameter, ink rheology and available extrusion pressure. To isolate the effect of structural porosity from intrastrand porosity, both bulk (cast) and printed structures were evaluated. In neat siloxane structures, printed in cross-ply (alternating 0° and 90° layered structures referred to as face-centered tetragonal or FCT^38^, Fig. 1d–g) and cast without microballoons, two regimes of compression response are observed. In the first regime (<40% ε in Fig. 2c), deformation is architecturally driven, accommodated by compaction and inlaying of upper layers in layers below. In the subsequent regime, deformation is driven by the strand material properties. The transition between the two will be referred to as structural “lock up”. This initial deformation mechanism of strand nestling in FCT printed structures has been investigated previously using *in situ* images acquired during compression loading and numerical simulations^7^.

Note that the inclusion of the T_g_44 microballoons minimally affects the first regime of response, yet substantially lowers the material stiffness in the second regime (above 40% strain). This is attributed to the T_g_44 microballoon compressibility, observed in cast resin filled with 40 vol% T_g_44 microballoons. The cast resin filled with 40 vol% T_g_113 microballoons is stiffer than the cast resin filled with 40 vol% T_g_44 microballoons, indicating that the T_g_113 microballoons are stiffer than the Tg44 microballoons. The bulk siloxane resin experiences comparatively little strain under applied loading, while cast siloxane filled with 40 vol% T_g_113 and 40 vol% T_g_44 microballoons undergoes 9% and 27% strain, respectively, under the same applied load. These strains are accommodated primarily through a nonlinear response at 1000 kPa and 300 kPa, respectively, attributed to yielding or breakage of the microballoons.

In the case of specimens cast with T_g_113 microballoons, we attribute the lower stiffness observed above 0.5 MPa to fracture of the glassy microballoon shell. In printed structures filled with 40 vol% T_g_113 microballoons, we observe a stiffer material response early on, leading us to conclude that, at 40 vol%, the glassy, rigid particles inhibit structural motion/strand nestling. This behavior is lessened in the 25 vol% T_g_113 printed structure, which exhibits greater deformation below 600 kPa. The strain accommodated during the first regime is reduced, in this case, from ~40% to 35%. For 25 vol% and 40 vol% loading T_g_113, the stiffness of the material above the lock-up stress (point at which structural porosity becomes very small) in the microballoon filled print is comparable to that of the neat resin print. Conversely, recall that the lower stiffness T_g_44 microballoons do not increase structural resistance to compression; in fact, they result in a lower stiffness response throughout loading beyond structural lock up or interlayer compaction. These results are presented to illustrate the effect of microballoon mechanical properties on open cell printed structures.

### Shape memory behavior

**Figure 3:**
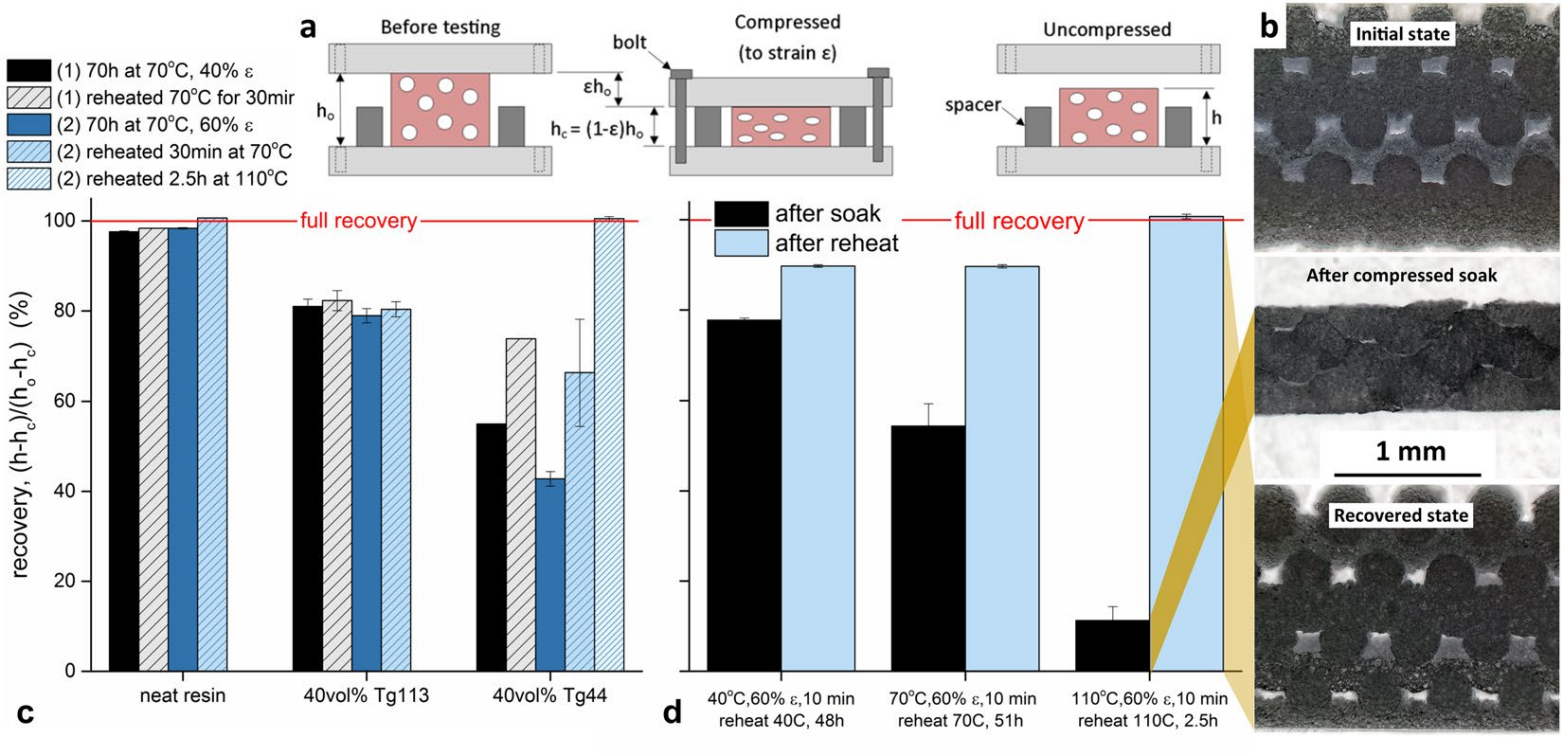
(**a**) Labeled schematic depicting the shape memory experiment, (**b**) optical microscopy images showing cross-sectional views of a printed structure with T_g_44 microballoons at different stages of the shape memory experiment, (**c**) shape memory behavior following thermal soak under compressive strain at 70 °C for 70 hours, and (**d**) time dependent recovery behavior in the T_g_44 microballoon prints near T_g_ and above T_g_.

Shape memory evaluations, quantified by compression set, were performed by holding printed structures under compression during thermal soak, cooling under compression and releasing the compressive load (Fig. 3a), were performed to assess the long term effect of microballoon addition on structural performance. Percent recovery was determined as a ratio of the recovered thickness to the compressive deformation. Neat siloxane prints exhibited a small but measurable compression set at 40% and 60% strain when held at 70 °C for 70 h (Fig. 3c). This thickness change was somewhat recoverable after reheating to 70 °C for 30 min. The addition of T_g_113 microballoons resulted in 20% reduction in thickness, following the same heating schedule at 40% and 60% strain. None of this deformation was recoverable after reheating to 70 °C for 30 min. The T_g_44 microballoons resulted in 45% and 57% reduction in thickness, following the same heating schedule at 40% and 60% strain, respectively. This material experienced noticeable recovery upon reheating, recovering 10–15% thickness at 70 °C for 30 min. Complete thickness recovery was observed upon reheating to 110 °C for 2.5 h.

The ability to retain a compressed shape after thermally soaking and cooling and then recover the original shape upon unconfined reheating is a property associated with shape memory polymers (SMPs)^39–41^. Shape recovery of the 40 vol% T_g_44 microballoon prints is dependent on soak and reheat temperatures (Fig. 3d). This effect is most pronounced at temperatures above the T_g_ of the microballoons. The shape recovery is evidenced by recovery of structural porosity and retention of microballoon spherical shape (Fig. 3b).

## Discussion

Elastomeric structures with shape memory behavior, accommodated by polymer shell microballoons were 3D printed and their influence on printability and structural performance was explored. Specifically, two different gas filled microballoons were selected to probe the effect of shell stiffness and T_g_ on compressive behavior and compression set in 3D printed structures. Introducing another level of porosity in the printed strands themselves enabled alteration of the mechanical response which was dependent on the microballoon shell stiffness. In the T_g_44 system, we observe significant compression set at short holds at temperatures above T_g_. While substantial recovery is observed at lower temperature reheats, complete structural recovery occurs upon reheating at elevated temperature (110 °C). This is attributed to re-expansion of the microballoons when heated above T_g_, with shape retention accommodated by the cross-linked structure. We observe reduced compression set in the higher T_g_ system, and a lack of recovery upon reheating. In this way, we illustrate the ability to tune structural response using a hierarchical combination of open and closed cell porosity, in conjunction with variable T_g_ microballoon addition. The shape memory behavior exhibited by the T_g_44 microballoon filled prints demonstrates their potential use in wearable protective padding and cushions with T_g_ optimized for human body temperature, in shape memory polymer matrices toward the development of tandem shape memory polymers capable of recovery in stages depending on the temperature^42, 43^. Future efforts to advance this work include multi-material printing and in-line mixing, to further tune structure, porosity and material properties.

## Methods

### Feedstock Materials

Polydimethylsiloxane resin (Dow Corning® SE 1700) with nanosilica filler for yield stress behavior and fracture toughness was used as the matrix phase in this study. The effect of microballoon shell material behavior on DIW structure compressibility was studied using two commercially available materials. Figure 1b depicts isobutane filled, thermally expanded poly(acrylonitrile-co-vinylidene chloride-co-methyl methacrylate) microballoons (AzkoNobel Expancel® 551 DE 40 d42) with a gas displacement density of 0.042 g/cc. These microballoons possess a glass transition temperature, T_g_, of 44 °C, measured using thermomechanical analysis (see Supplementary Figure 1a), rather than dynamic scanning calorimetry due to their extremely light weight. Figure 1c shows phenol formaldehyde resin shell microballoons (Asia Pacific Microballoons BJO-0930) with a liquid displacement density of 0.21–0.25 g/cc. Phenolic microballoons possess a T_g_ of 113 °C, measured using dynamic scanning calorimetry (see Supplementary Figure 1b).

Particle cross-sectional areas were measured using automated transmitted light microscopy (Malvern Morphologi G3) and the particle size distribution was calculated assuming the particles are spherical. Hence, we report a circular effective diameter value in Fig. 1a. Despite the difference in particle size distribution between the two microballoon lots, shell thickness for each was measured to be 1–2 μm using scanning electron microscopy and optical microscopy of fractured particles. Note that there are a significant fraction of doublets and triplets in the T_g_113 system, as opposed to the T_g_44 system.

### DIW Ink Preparation

A volumetric loading of 40% microballoons in the silica filled siloxane resin was used as a standard to compare the two microballoon materials. This selection was made on the basis that 40 vol% filler content should not result in significant jamming and thickening behavior given the broad size distributions^44^, yet it should have a significant impact on the overall mechanical performance of printed structures.

Resin was prepared by blending 40 vol% microballoons into the siloxane base resin (SE 1700 Part A base) using a vacuum gravitational mixer (Thinky ARV 310) at 2000 rpm for 1 min. After this time, the microballoon resin mixture was hand mixed, followed by another round of non-contact mixing under vacuum at 2000 rpm for 1 min. While no noticeable heating occurred during blending, the material was allowed to cool in a standing water bath for 5 min, prior to non-contact mixing of SE1700 Part B curing agent at 2000 rpm for 20 s. The microballoon suspension was transferred to a 30 cc syringe for printing.

### Ink Rheology

The effect of microballoon addition on the rheological behavior of siloxane resin was evaluated using rotational rheology (TA Instruments AR 2000ex equipped with cross-hatched parallel plates to prevent microballoon compression and mitigate wall slip effects). Oscillatory experiments (Fig. 2a) were performed at 10 Hz and power law behavior was observed under continuous flow (Fig. 2b).

### DIW Printing

Printing was performed using a displacement controlled 3-axis 3D printing platform, resulting in cross-ply structures with each subsequent layer fashioned at a 90° angle from the one prior. This structure is referred to as face-centered tetragonal (FCT) and is discussed in detail by Maiti *et al*.^45^. A 250 µm nozzle was used to produce these prints, which were 50 × 50 mm squares at 8 layers thick. Printed structures were oven cured under a nitrogen purge for 6 h at 60 °C, followed by 1 h at 150 °C and a post cure at 125 °C for 12 h. These structures possessed overall densities of 0.50 g/cc and 0.42 g/cc using 40 vol% of the T_g_113 and T_g_44 microballoons, respectively.

### Thermal and Mechanical Characterization

Mechanical response was evaluated under compressive loading supplied by a Instron 5944 universal testing frame equipped with a 2 kN load cell. Strain was measured using a 0.5″ strain gage extensometer on 2.2 cm (7/8 in) diameter, 1.6 mm thick printed FCT specimens. Compression set was assessed on printed FCT structures punched to 13 mm diameter and 1.6 mm thick (8 layers). Following ASTM D395, printed structures were compressed to 40% and 60% strain and soaked at 70 °C for 70 h, cooled under compression, released and allowed to relax for 10 min, prior to measurement. To evaluate recovery, specimens were reheated at temperatures equal to or greater than that of the compressed soak for a series of times until no further structural changes were observed.

## Electronic supplementary material

Microballoon glass transition temperatures
