# Supplementary material for: 3D Printed Silicones with Shape Memory
Source: Sci Rep. 2017 Jul 5;7:4664. doi: 10.1038/s41598-017-04663-z (PMC5498669; doi:10.1038/s41598-017-04663-z)
Supplement: Supplementary file 1 — Microballoon glass transition temperatures [file 41598_2017_4663_MOESM1_ESM.doc]

Supporting Information

3D Printed Silicones with Shape Memory

**Amanda S. Wu^1^,*, Ward Small IV^2^, Taylor M. Bryson^1^, Emily Cheng^1^, Thomas R. Metz^1^, Stephanie E. Schulze^3^, Eric B. Duoss^1^ and Thomas S. Wilson^2^**

^1^Lawrence Livermore National Laboratory, Materials Engineering Division, Livermore, CA 94550, USA

^2^Lawrence Livermore National Laboratory, Materials Science Division, Livermore, CA 94550, USA

^3^Department of Energy’s National Security Campus, managed by Honeywell, Materials Engineering, Kansas City, MO 64147, USA

*wu36@llnl.gov


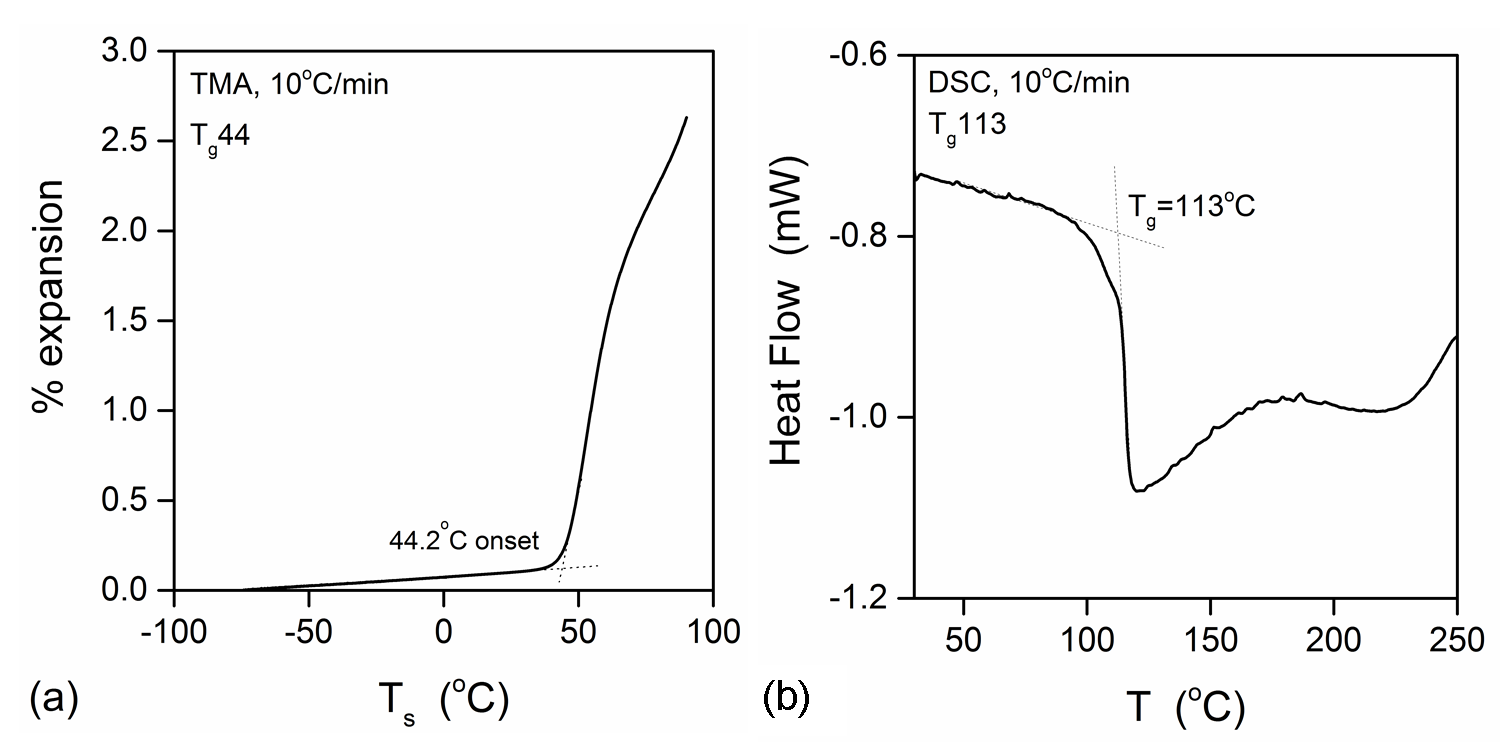


**Figure S1.** Thermomechanical behavior of (a) T_g_44 microspheres (131°C T_m_) assessed by TMA and (b) T_g_113 microspheres assessed by DSC
